# Metabolic Differentiation of Co-occurring Accumulibacter Clades Revealed through Genome-Resolved Metatranscriptomics

**DOI:** 10.1128/mSystems.00474-21

**Published:** 2021-07-06

**Authors:** Elizabeth A. McDaniel, Francisco Moya-Flores, Natalie Keene Beach, Pamela Y. Camejo, Ben O. Oyserman, Matthew Kizaric, Eng Hoe Khor, Daniel R. Noguera, Katherine D. McMahon

**Affiliations:** a Department of Bacteriology, University of Wisconsin—Madison, Madison, Wisconsin, USA; b Department of Civil and Environmental Engineering, University of Wisconsin—Madison, Madison, Wisconsin, USA; c Geroscience Center for Brain Health and Metabolism (GERO), Santiago, Chile; d Millenium Initiative for Collaborative Research on Bacterial Resistance (MICROB-R), Iniciativa Científica Milenio, Santiago, Chile; e Millennium Institute for Integrative Biology (iBio), Santiago, Chile; f Carollo Engineers, Inc., Broomfield, Colorado, USA; g Bioinformatics Group, Wageningen University and Research, Wageningen, The Netherlands; h Microbial Ecology, Netherlands Institute of Ecological Research, Wageningen, The Netherlands; i DOE Great Lakes Bioenergy Research Center, Madison, Wisconsin, USA; j Microbiology Doctoral Training Program, University of Wisconsin—Madison, Madison, Wisconsin, USA; University of Waterloo

**Keywords:** EBPR, environmental microbiology, genomics, metagenomics, metatranscriptomics

## Abstract

Natural microbial communities consist of closely related taxa that may exhibit phenotypic differences and inhabit distinct niches. However, connecting genetic diversity to ecological properties remains a challenge in microbial ecology due to the lack of pure cultures across the microbial tree of life. “*Candidatus* Accumulibacter phosphatis” (Accumulibacter) is a polyphosphate-accumulating organism that contributes to the enhanced biological phosphorus removal (EBPR) biotechnological process for removing excess phosphorus from wastewater and preventing eutrophication from downstream receiving waters. Distinct Accumulibacter clades often coexist in full-scale wastewater treatment plants and laboratory-scale enrichment bioreactors and have been hypothesized to inhabit distinct ecological niches. However, since individual strains of the Accumulibacter lineage have not been isolated in pure culture to date, these predictions have been made solely on genome-based comparisons and enrichments with varying strain compositions. Here, we used genome-resolved metagenomics and metatranscriptomics to explore the activity of coexisting Accumulibacter strains in an engineered bioreactor environment. We obtained four high-quality genomes of Accumulibacter strains that were present in the bioreactor ecosystem, one of which is a completely contiguous draft genome scaffolded with long Nanopore reads. We identified core and accessory genes to investigate how gene expression patterns differed among the dominating strains. Using this approach, we were able to identify putative pathways and functions that may confer distinct functions to Accumulibacter strains and provide key functional insights into this biotechnologically significant microbial lineage.

**IMPORTANCE** “*Candidatus* Accumulibacter phosphatis” is a model polyphosphate-accumulating organism that has been studied using genome-resolved metagenomics, metatranscriptomics, and metaproteomics to understand the EBPR process. Within the Accumulibacter lineage, several similar but diverging clades are defined by the shared sequence identity of the polyphosphate kinase (*ppk1*) locus. These clades are predicted to have key functional differences in acetate uptake rates, phage defense mechanisms, and nitrogen-cycling capabilities. However, such hypotheses have largely been made based on gene content comparisons of sequenced Accumulibacter genomes, some of which were obtained from different systems. Here, we performed time series genome-resolved metatranscriptomics to explore gene expression patterns of coexisting Accumulibacter clades in the same bioreactor ecosystem. Our work provides an approach for elucidating ecologically relevant functions based on gene expression patterns between closely related microbial populations.

## INTRODUCTION

Naturally occurring microbial assemblages are composed of closely related species or subpopulations and harbor extensive genetic diversity. Coherent lineages are often comprised of distinct clades, ecotypes, or “backbone subpopulations” defined by high within-group sequence similarities that translate to ecologically relevant diversity (1–3). For example, genetically diverse ecotypes of the marine cyanobacterium *Prochlorococcus* that are 97% identical by their 16S rRNA gene sequences exhibit markedly different light-dependent physiologies and distinct seasonal and geographical patterns (1, 2, 4, 5). Closely related *Nitrospira* strains representing species-like lineages that coexisted for long periods of time in a full-scale wastewater treatment plant exhibited functional differences in preferences for nitrite concentrations and carbon substrates (6). However, dissecting the emergent ecological properties of species-like coherent microbial lineages is still a grand challenge in microbial ecology given that these principles are likely not universal among the breadth of microbial diversity and is further hindered when there is a lack of pure cultures (7–9).

Enhanced biological phosphorus removal (EBPR) is an economically and environmentally significant process for removing excess phosphorus from wastewater. This process depends on polyphosphate-accumulating organisms (PAOs) that polymerize inorganic phosphate into intracellular polyphosphate. “*Candidatus* Accumulibacter phosphatis” (referred to here as Accumulibacter) is a member of the *Betaproteobacteria* in the *Rhodocyclaceae* family and has long been a model PAO (10, 11). Accumulibacter achieves net phosphorus removal through cyclical anaerobic-aerobic phases of feast and famine. In the initial anaerobic phase, abundantly available volatile fatty acids (VFAs) such as acetate are converted to polyhydroxyalkanoates (PHAs), carbon storage polymers. However, PHA formation requires significant ATP input and reducing power, which is supplied through polyphosphate and glycogen degradation. In the subsequent aerobic phase in which oxygen is available as a terminal electron acceptor and soluble carbon sources are depleted, Accumulibacter uses PHA reserves for growth. Accumulibacter recovers energy during aerobic PHA degradation by replenishing polyphosphate and glycogen reserves, which can be later used for future PHA formation as the anaerobic/aerobic cycle repeats. This feast-famine oscillation through sequential anaerobic-aerobic cycles operating on minute-to-hourly time scales gives rise to net phosphorus removal and the overall EBPR process (10, 12). A few ‘omics-based studies have revealed potential regulatory modules that could coordinate Accumulibacter’s complex physiological behavior under these very dynamic environmental conditions (13–16), but few have attempted to assign unique gene expression dynamics to specific clades (17).

Accumulibacter can be subdivided into two main types (types I and II) and further subdivided into multiple clades based upon polyphosphate kinase (*ppk1*) sequence identity since the 16S rRNA marker is too highly conserved among the breadth of known Accumulibacter lineages to resolve species-level differentiation (18–21). The two main types share approximately 85% nucleotide identity across the *ppk1* locus (21), which mirrors genome-wide average nucleotide identity (ANI) (22). The persistence of several phylogenetically distinct clades within the Accumulibacter lineage in both full-scale wastewater treatment plants and laboratory-scale enrichment bioreactors suggests that different clades may inhabit distinct ecological niches (17, 23). This is largely supported by comparative genomics of Accumulibacter metagenome-assembled genomes (MAGs) recovered from different bioreactor systems and classified using the *ppk1* locus (14, 17, 22, 24–27). Members of different clades have been hypothesized to vary in their acetate uptake rates, nitrate reduction capacities, and phage defense mechanisms (22, 26). For example, clade IA seems to exhibit higher acetate uptake rates and phosphate release rates, whereas evidence exists that clade IIA can reduce nitrate but that clade IA cannot (25, 28). However, the full breadth of ecological differentiation among Accumulibacter clades is not yet clear.

Here, we used genome-resolved metagenomics and time series metatranscriptomics to investigate gene expression patterns of coexisting Accumulibacter strains (representing two main clades) during a standard EBPR cycle. We assembled four high-quality genomes of the dominant (clades IA and IIC) and low-abundance (clades IIA and IIF) Accumulibacter strains present in the bioreactor ecosystem. Additionally, we constructed a contiguous draft genome of an Accumulibacter clade IIC genome achieved using long Oxford Nanopore technology (ONT) reads, representing one of the highest-quality genomes for this clade recovered to date. Using these assembled genomes, we performed time series RNA sequencing (RNA-seq) throughout a typical EBPR feast-famine cycle to study the expression profiles of different Accumulibacter strains, with an emphasis on core and flexible gene contents. More generally, our work reveals putative ecological functions of coexisting taxa within a dynamic ecosystem.

## RESULTS

### Enrichment of coexisting Accumulibacter clades.

**FIG 1:**
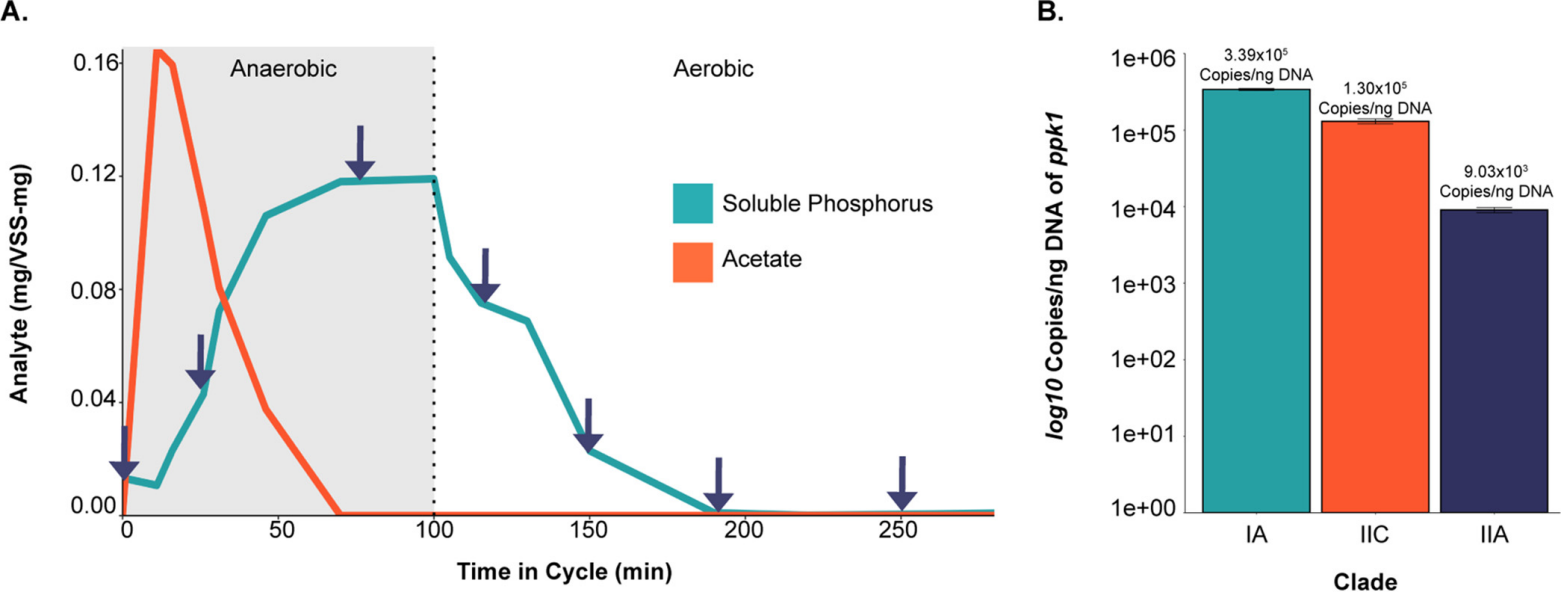
Overview of the experimental design and enriched Accumulibacter clades. (A) Overview of the EBPR cycle and sampling scheme. Samples were collected for RNA sequencing over the course of a normal EBPR cycle, with reported measurements for soluble phosphorus and acetate normalized by volatile suspended solids (VSS). Samples were taken at the 0-, 31-, 70-, 115-, 150-, 190-, and 250-min time points. (B) Abundances of different Accumulibacter clades by *ppk1* copies per nanogram of DNA. Quantification of Accumulibacter clades was performed by qPCR of the *ppk1* gene as described previously by Camejo et al. (23).

We inoculated a 2-liter sequencing-batch reactor with activated sludge from a full-scale wastewater treatment plant in Madison, WI, and maintained operation for 40 months. The reactor was primarily fed with acetate and operated under cyclic anaerobic-aerobic phases with a 4-day solids retention time to simulate the EBPR process and enrich for Accumulibacter (Fig. 1). During a period of stable EBPR (14 months following inoculation) in which acetate was completely diminished by the start of the aerobic phase and soluble phosphate was below 1.0 mg liter^−1^ at the end of the aerobic phase, we collected seven samples across a single cycle (three in the anaerobic phase and four in the aerobic phase) for RNA sequencing (Fig. 1A). We performed quantitative PCR (qPCR) of *ppk1* as described previously (23) to quantify Accumulibacter clades present in the reactor on the same day as the RNA-seq experiment (Fig. 1B). At the time of the RNA-seq experiment, the bioreactor was primarily enriched in Accumulibacter clade IA, followed by clade IIC and clade IIA.

**FIG 2:**
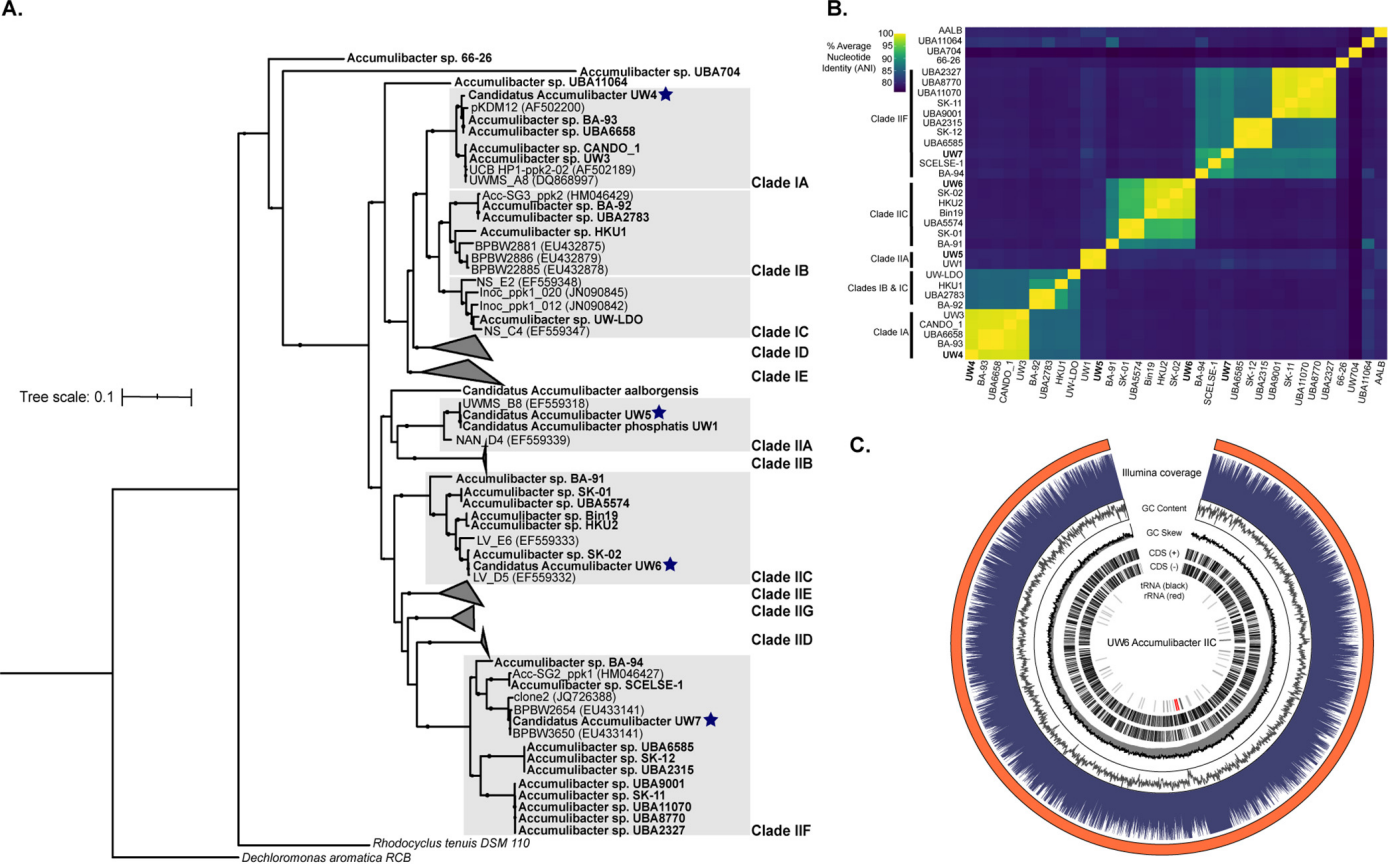
Assembly of four high-quality Accumulibacter genomes. (A) Phylogenetic tree of *ppk1* nucleotide sequences from available Accumulibacter reference genomes, clone sequences, and the four assembled Accumulibacter genomes from this study. Tips in bold represent *ppk1* sequences from metagenome-assembled genomes, whereas others are sequences from clone fragments available under the indicated GenBank accession numbers. Starred tips represent *ppk1* sequences from the four assembled Accumulibacter genomes from this study. The tree was constructed using RAxML with 100 rapid bootstraps. (B) Pairwise genome-wide ANI comparisons of all publicly available Accumulibacter references and the four new Accumulibacter genomes, denoted in boldface type. Genomes are grouped according to clade designation by *ppk1* sequence identity and in order of the *ppk1* phylogeny, except for those that do not fall in an established clade. (C) Genome diagram of the UW6 Accumulibacter clade IIC MAG (98.6% completeness, 2.5% contamination, and 5.18 Mbp). Layers represent, from top to bottom, coverage over 1,000-bp windows, GC content and skew across the same 1,000-bp windows, coding sequences (CDS) on the positive and negative strands, and positions of predicted rRNA and tRNA sequences.

From metagenome samples collected from this bioreactor previously, we assembled high-quality genomes belonging to four different Accumulibacter clades (Table 1), including the dominant clades IA and IIC (strains IA-UW4 and IIC-UW6, respectively) and the less abundant clades IIA and IIF (strains IIA-UW5 and IIF-UW7, respectively) (Fig. 2). Each of the assembled genomes falls within the established Accumulibacter clade nomenclature as defined by *ppk1* sequence identity and phylogenetic placement compared to other publicly available Accumulibacter genomes and clone sequences (Fig. 2A). Additionally, the *ppk1* hierarchical structure is also reflected by pairwise average nucleotide identity (ANI) boundaries between clades, with some exceptions for newly assembled Accumulibacter genomes that fall outside the established “*Ca.* Accumulibacter phosphatis” lineage (Fig. 2B). All assembled genomes are above 90% complete and contain <5% redundancy as calculated by CheckM (29) (Table 1). We were able to scaffold the assemblies of strains IIA-UW5 and IIC-UW6 using long Nanopore reads, constructing a completely contiguous draft genome of the IIC-UW6 strain. To our knowledge, the assembled IIC-UW6 genome is the most contiguous, highest-quality reference genome available for this clade, providing a valuable new Accumulibacter reference genome (Fig. 2C).

**TABLE 1:** Assembled Accumulibacter genome statistics^*a*^

Genome	% completion	% contamination	Size (Mbp)	No. of contigs	% GC content
IIA-UW5	98.99	5.24	4.88	68	64.3
IIC-UW6	98.57	2.46	5.18	1	61.1
IIF-UW7	98.97	3.04	4.87	102	66.3
IA-UW4	92.9	3.37	4.29	358	64

a Genome quality calculations for completeness and contamination were made with CheckM based on the presence/absence of single-copy genes (29).

### Distribution of shared and flexible gene contents between clades.

**FIG 3:**
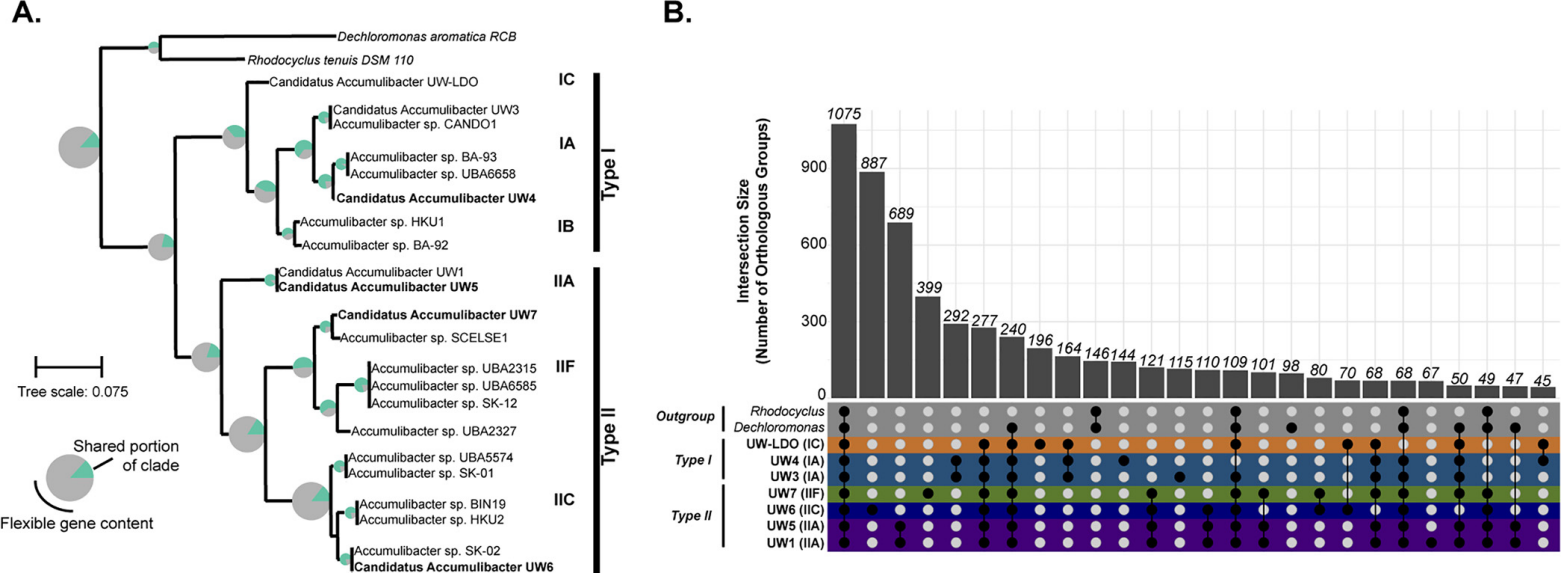
Core and flexible gene contents among the Accumulibacter lineage and UW genomes. (A) Phylogenetic tree of the coding regions for the *ppk1* locus among high-quality Accumulibacter references, UW genomes, and the outgroup taxa Dechloromonas aromatica RCB and Rhodocyclus tenuis DSM 110. The pie graph at each node represents the fraction of genes that are shared and flexible for genomes belonging to that clade. (B) Upset plot representing the intersections of groups of orthologous gene clusters among UW genomes and outgroups, analogous to a Venn diagram. A total of 1,075 gene families were shared among all UW Accumulibacter clades and outgroup genomes, and 277 separate gene families were found only within the UW Accumulibacter clades. Each bar represents the number of orthologous gene clusters, and the dot plot represents the genomes in which the groups intersect.

We characterized the functional diversity of different Accumulibacter clades through clustering of orthologous groups of genes (COG) (Fig. 3A). We repeated a COG analysis reported previously by Skennerton et al. (26) and Oyserman et al. (30) but included an updated set of high-quality Accumulibacter references, the University of Wisconsin (UW)-generated genomes, and the outgroups Dechloromonas aromatica and Rhodocyclus tenuis (Fig. 3). The Accumulibacter lineage exhibits a tremendous amount of functional diversity, with only approximately 25% of COGs shared among all Accumulibacter clades (Fig. 3A). Additionally, there is extensive diversity within the two Accumulibacter types and within individual clades, with only a few representatives of each clade being more than ∼75% similar to each other by gene content (see Fig. S1 in the supplemental material). Specifically, clade IIC broadly harbors the most diversity of all sampled genomes by gene content, with representatives of this clade not sharing more than 25% of COGs (Fig. 3A). Other clades are more similar by gene content, but this could be due to fewer genomes sampled from these clades. Most of the highest-quality, publicly available Accumulibacter genome references belong to clades IIF and IIC.

10.1128/mSystems.00474-21.1 FIG S1 Pairwise Pearson correlation coefficients of shared gene content as a measure of how similar each of the UW genomes is to each other based on COGs. Download FIG S1, TIF file, 0.3 MB. Copyright © 2021 McDaniel et al. 2021 McDaniel et al. https://creativecommons.org/licenses/by/4.0/ This content is distributed under the terms of the Creative Commons Attribution 4.0 International license.

We then compared COGs between Accumulibacter clades collected from bioreactors seeded from the Nine Springs Wastewater Treatment Plant in Madison, WI, as these genomes represent a shared origin (Fig. 3B). In addition to two laboratory-scale bioreactors harboring distinct Accumulibacter clades (strains UW1 and UW3 and strain UW4-7, respectively) (22, 24), we recently characterized clade IC (strain UW-LDO-IC) using genome-resolved metagenomics and metatranscriptomics (14). Overall, genomes in type I and type II cluster, respectively, where genomes within type I are overall more similar to each other, as expected (Fig. S1). Clade IIA genomes (strains UW1 and UW5) were assembled from separate enrichments derived from the same full-scale treatment plant approximately 12 years apart but are very similar by COG presence/absence (Fig. S1). UW genomes within type II are less similar by gene content and seem to be more diverse in this regard, although again, this could be due to undersampling of type I (8 genomes in type I and 14 in type II). Additionally, clade IIF contains more overlap in gene content with type I genomes in gene families than some type II genomes do with each other (Fig. S1).

COG overlap and uniqueness are shown by the intersection of COGs between UW Accumulibacter clades and the *Rhodocyclus* and *Dechloromonas* outgroups (Fig. 3B). There are 1,075 gene families shared among all UW Accumulibacter clades and outgroup genomes, with 277 separate gene families only within the UW Accumulibacter clades (Fig. 3B). Among individual clades, there are 887 groups only within clade IIC (UW6), 399 within clade IIA (UW1 and UW5), 399 within clade IIF (UW7), 292 within clade IA (UW3 and UW4), and 196 within clade IC (UW-LDO). Although clade IIC strain UW6 contains more overlap in gene content with SK-02 (Fig. 3A), the intersection analysis (Fig. 3B) identified gene families that are available to individual strains within the same bioreactor ecosystem, highlighting the potential for functional differentiation.

### Transcriptional profiles of Accumulibacter strains.

**FIG 4:**
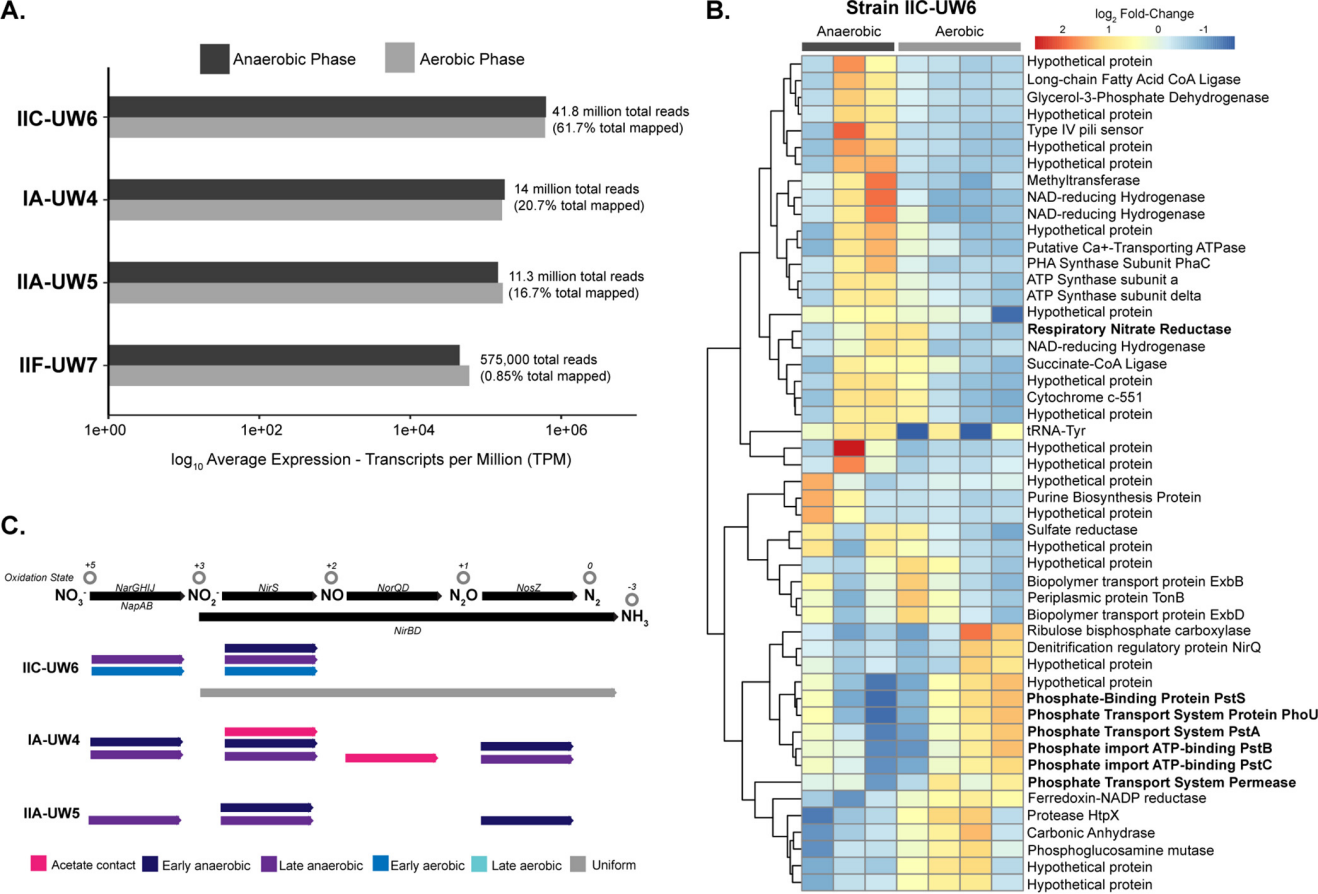
Transcriptomic profiles of Accumulibacter clades. (A) Average expression profiles of each reference genome in the anaerobic and aerobic phases. The IIC-UW6 genome mapped the most transcripts, even though IA-UW4 was more abundant. Reads were competitively mapped to the four assembled Accumulibacter clade genomes and normalized by transcripts per million. The sums of the normalized counts of the three samples in the anaerobic phase and four samples in the aerobic phase were averaged, respectively, and plotted on a log_10_ scale. Numbers and percentages adjacent to normalized bars represent the raw numbers of reads that are mapped to each genome. (B) Top 50 differentially expressed genes in clade IIC-UW6. All 50 genes pass the threshold of a ±1.5-log_10_ fold change across the cycle. Annotations were predicted from a combination of Prokka (69) and KofamKOALA (70). (C) Denitrification gene expression among clades IIC-UW6, IA-UW4, and IIA-UW5. We summarized expression for genes involved in denitrification by highlighting parts of the cycle in which certain genes are expressed the most. This is characterized by acetate contact, early anaerobic, late anaerobic, early aerobic, and late aerobic, where uniform describes uniform expression across the entire cycle. If an arrow is missing for a step, that particular clade did not contain confident annotations within the genome for those genes. For nitrate reduction, the *nar* system is represented as the top arrow, and the *nap* system is represented as the bottom arrow in the first column. For subsequent steps, multiple arrows are shown for a single step in which particular genes were highly expressed in multiple phases.

Although *ppk1* locus quantification showed that the bioreactor was mostly enriched in clade IA (Fig. 1B), surprisingly, more RNA-seq reads mapped to the clade IIC-UW6 genome than to the clade IA-UW4 genome (Fig. 4A). Approximately 40 million total transcriptional reads aligned to the strain IIC-UW6 genome across the full time series experiment, whereas strains IA-UW4 and IIA-UW5 recruited roughly the same number of reads, with 14 million and 11 million, respectively (Table 2). Markedly lower read levels mapped to the IIF-UW7 strain than to the other three clades, and therefore, we do not include the IIF-UW7 strain in our subsequent analyses. We explored the top differentially expressed genes in strain IIC-UW6, comparing anaerobic and aerobic conditions (Fig. 4B). Genes upregulated in the anaerobic phase belonged to pathways for central-carbon transformations, fatty acid biosynthesis, and PHA synthesis. Interestingly, all genes within the high-affinity phosphate transport system Pst were upregulated in the aerobic phase. These include the substrate-binding component *pstS*, the *phoU* regulator, and the ABC-type transporter *pstABC* complex (Fig. 4B).

**TABLE 2:** Metatranscriptomic reads mapped to Accumulibacter genomes^*a*^

Clade	No. of reads							
	Anaerobic-1045	Anaerobic-1116	Anaerobic-1155	Aerobic-1240	Aerobic-1315	Aerobic-1355	Aerobic-1455	Total
UW6-IIC	5,226,723.91	5,892,548.407	6,755,519.231	7,902,549.247	5,004,518.353	6,914,955.951	4,132,968.794	41,829,783.89
UW4-IA	1,484,341.048	2,216,299.172	2,290,440.732	3,362,431.038	1,950,258.638	1,558,604.931	1,157,344.685	14,019,720.24
UW5-IIA	1,063,676.087	1,357,479.512	1,706,229.277	2,197,556.23	1,763,358.606	1,171,469.831	2,065,179.234	11,324,948.78
UW7-IIF	51,037.95551	78,248.90971	84,519.76084	121,226.4853	86,070.40356	90,567.28675	63,866.28633	575,537.088

a Counts of raw metatranscriptomic reads mapped to each Accumulibacter genome with kallisto (88) in each sample for the three anaerobic samples and four aerobic samples and total reads mapped for all samples are shown. The number in each construct refers to the time point during the cycle that samples were taken. Anaerobic-1045 refers to the sample taken during the anaerobic phase at 10:45 AM (corresponds to the time in cycle [min] in Fig. 1A).

The coexisting clades in the bioreactor system differ in both the presence of sets of nitrogen-cycling genes and their expression profiles (Fig. 4C). In strain IA-UW4, we detected the *napAGH* genes for nitrate reduction, whereas we did not detect *napB*, possibly due to these genes being close to the end of a contig in this genome. We also detected a *nirS*-like nitrite reductase, both subunits of the nitric oxide reductase *norQD*, and the nitrous oxide reductase *nosZ* in strain IA-UW4. Most of these genes in strain IA-UW4 exhibit the highest-expression patterns in the early and late anaerobic stages (Fig. 4C). Conversely, strain IIC-UW6 contains the *narGHIJ* machinery for nitrate reduction and a different *nirS-*like nitrite reductase than that in IA-UW4 and does not contain a nitrous oxide reductase. However, strain IIC-UW6 contains the nitrite reductase *nirBD* for reducing nitrite to ammonium, which is uniformly expressed across the cycle (Fig. 4C).

### Expression dynamics of genes shared by strains IA-UW4 and IIC-UW6.

**FIG 5:**
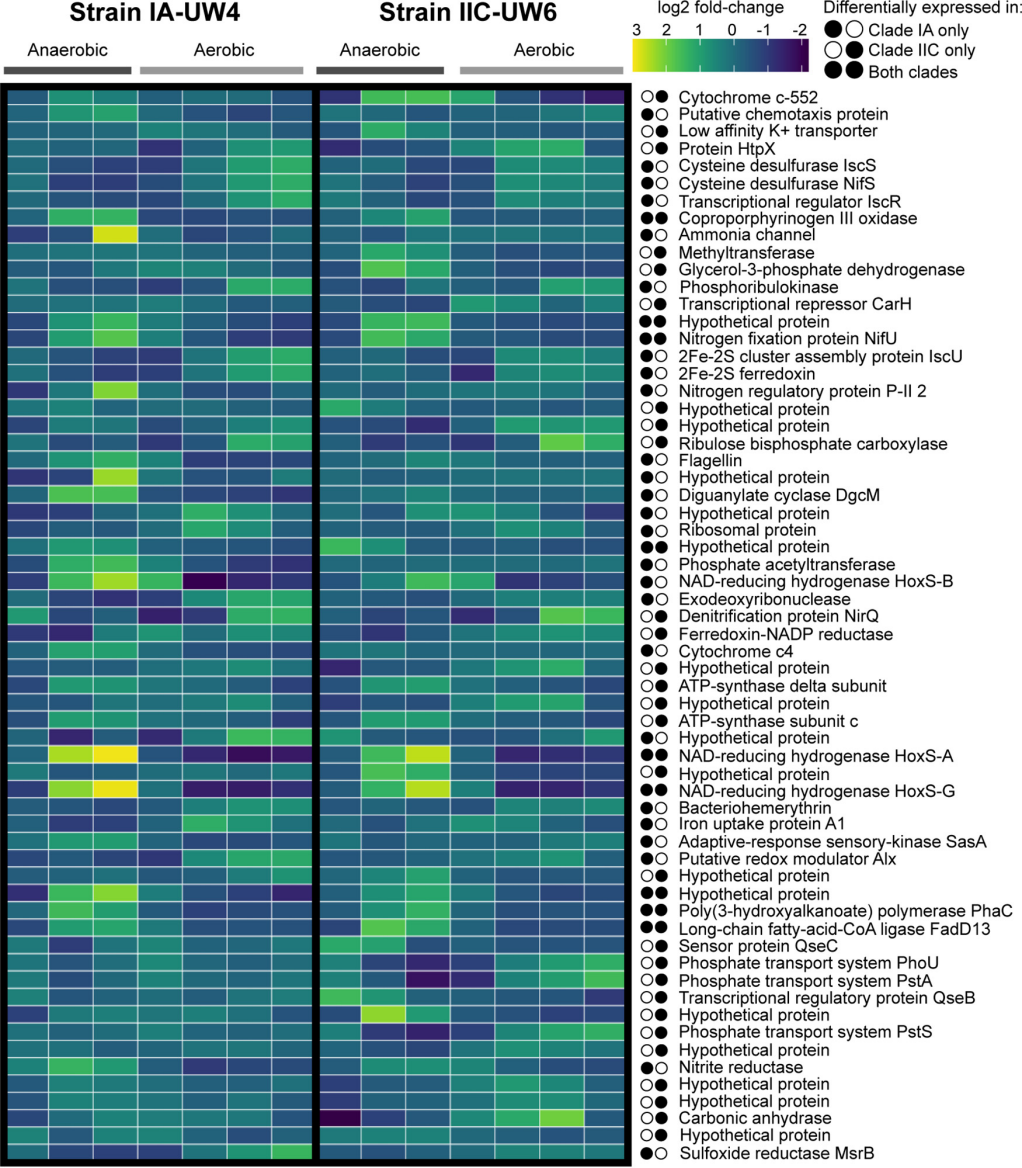
Differential expression of shared genes in strains IA-UW4 and IIC-UW6. Gene expression profiles of core COGs across the EBPR cycle that are differentially expressed between anaerobic and aerobic conditions in either IA-UW4, IIC-UW6, or both strains are shown. To consider a gene differentially expressed in a strain, the COG must exhibit a ±1.5-fold change in expression between anaerobic and aerobic phases. Homologs that are directly compared to each other in IA-UW4 and IIC-UW6 are orthologous genes that belong to the same cluster.

When characterizing the expression dynamics of COGs shared between strains IA-UW4 and IIC-UW6, we found very few orthologs that exceeded the 1.5-fold change cutoff for differential expression in both strains (Fig. 5). These genes include a coproporphyrinogen III oxidase, NAD-reducing HoxS subunits, the *phaC* subunit of the PHA synthase, the long-chain-fatty-acid CoA ligase FadD13, and several hypothetical proteins. The fatty acid CoA ligase incorporates ATP, CoA, and fatty acids of various lengths to form acyl-CoA to be degraded for energy production, incorporated into complex lipids, or used in other metabolic pathways (31). The acyl-CoA ligase is upregulated in the anaerobic phase of both clades, suggesting a role in anaerobic carbon metabolism, as acyl-CoA can ultimately form acetyl-CoA through beta-oxidation. The *phaC* subunit of the PHA synthase is differentially expressed in the anaerobic phase for both clades, as is typical of the hallmark EBPR metabolism for Accumulibacter (13, 32).

Additionally, both the IA-UW4 and IIC-UW6 strains carry most of the subunits for the bidirectional NiFe Hox system, encoding an anaerobic hydrogenase. The HoxH and HoxY beta and delta subunits form the hydrogenase moiety, and the FeS-containing HoxF and HoxU alpha and gamma subunits catalyze NAD(P)H/NAD(P)^+^ oxidation/reduction coupled to the hydrogenase moiety (33–35). In both clades, the FeS cluster HoxF and HoxU alpha and gamma subunits are strongly differentially expressed in the anaerobic phases, as was observed previously in a genome-resolved metatranscriptomic investigation of strain IIA-UW1 (13). However, the IIC-UW6 genome is missing the HoxY delta subunit of the hydrogenase moiety, and only strain IA-UW4 exhibits differential expression of the HoxH beta subunit in the anaerobic phase. Strain IIC-UW6 exhibits a higher expression level of HoxH in the late anaerobic and early aerobic phases but does not meet the threshold cutoff for differential gene expression. A previous study focused on strain IIA-UW1 also demonstrated hydrogenase activity in the anaerobic phase and then demonstrated hydrogen gas production after acetate addition (13). The hydrogen gas production was hypothesized to replenish NAD^+^ after glycogen degradation. A subsequent comparative genomics analysis suggested that the Hox system is an ancestral state of Accumulibacter (30). Furthermore, recent metabolic models that integrated hydrogen production under periods of excess acetate or glycogen were better able to capture the diversity of observed EBPR stoichiometry (36). In this ecosystem, both strains may exhibit anaerobic hydrogenase activity and, thus, produce hydrogen, but due to the missing subunit in the IIC-UW6 genome and the lack of differential gene expression, this is uncertain.

Interestingly, only one strain exhibited differential expression patterns of several core ortholog subsets important for EBPR-related functions. For example, subunits of the high-affinity phosphate transporter Pst system are differentially expressed only in strain IIC-UW6 and not IA-UW4, as observed for strain IIC-UW6 as described above (Fig. 4B). Additionally, nitrogen-cycling genes that contain orthologs in both clades display different expression profiles. A nitrite reductase is differentially expressed only in the anaerobic phase in strain IA-UW4, and the denitrification protein NirQ is differentially expressed only in strain IIC-UW6, whereas the nitrogen fixation protein subunit NifU is differentially expressed in both strains. To activate acetate to acetyl-CoA, the high-affinity acetyl-CoA synthase (ACS) or low-affinity acetate kinase/phosphotransacetylase (AckA/Pta) pathways can be employed. The strain IA-UW4 assembly is missing an acetate kinase, and phosphate acetyltransferase is differentially expressed only in this strain but not in IIC-UW6. Within strain IIC-UW6, both the acetate kinase and phosphate acetyltransferase genes exhibit relatively low levels of expression over the time series. Both strains contain several acetyl-CoA synthases that are highly expressed in the anaerobic phase, particularly upon acetate contact, but do not exhibit notable differences in differential expression between the two phases. Previous work based on community proteomics and enzymatic assays suggested that although both activation pathways are present and expressed in Accumulibacter, the high-affinity pathway is preferentially used (37, 38). Although a phosphate acetyltransferase is differentially expressed in strain IA-UW4, this gene has lower transcript levels than the acetyl-CoA synthase, providing more evidence that the lineage (specifically both clades IA and IIC) may employ the high-affinity system.

### Differential expression of flexible genes in strain IA-UW4.

**FIG 6:**
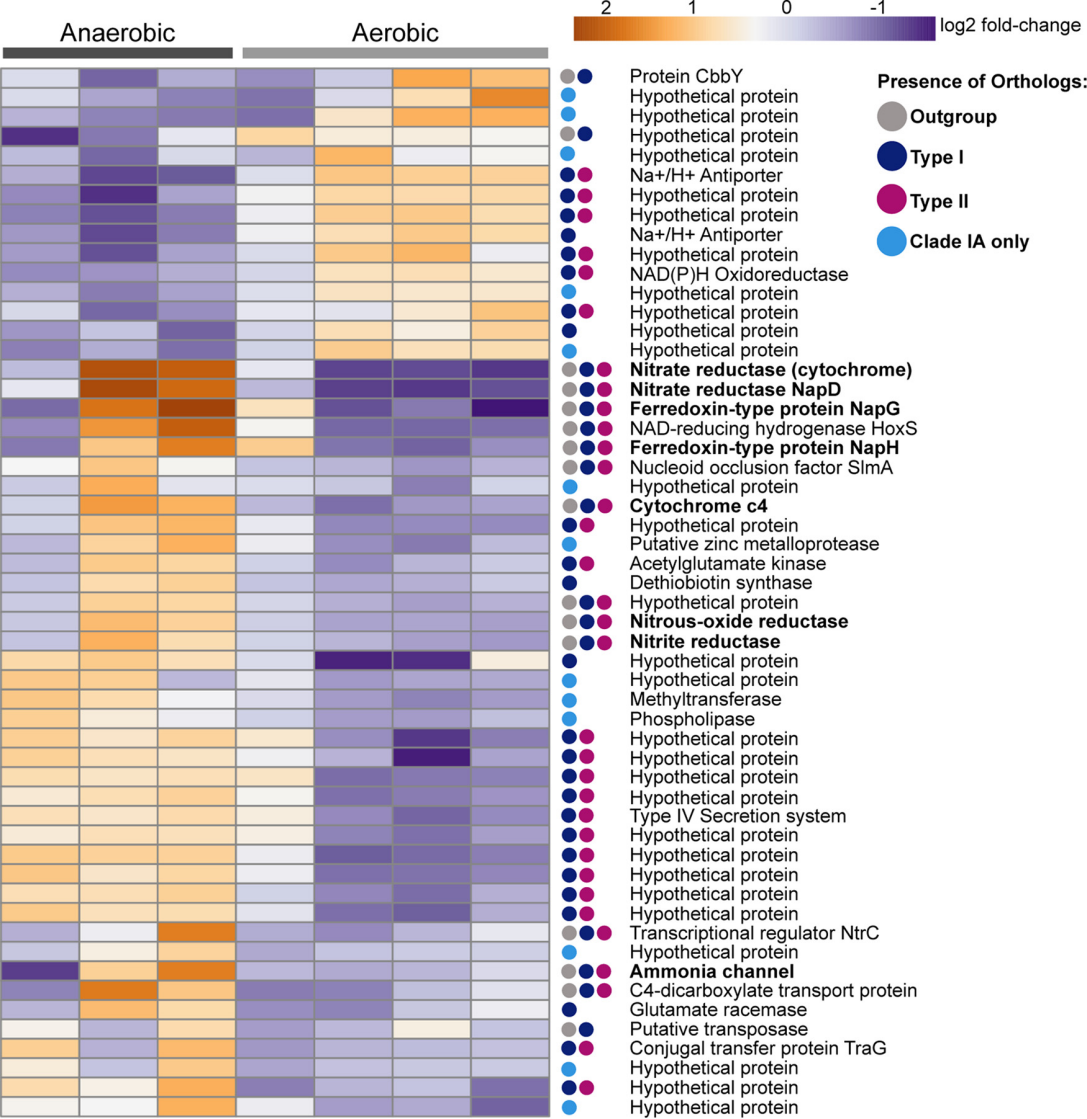
Differential expression of accessory genes in strain IA-UW4. Gene expression profiles of groups of accessory genes in strain IA-UW4 that are not present in the IIC-UW6 genome are shown. A gene was considered differentially expressed between the anaerobic and aerobic conditions if it exhibited greater or less than a ±1.5-fold change in expression. Colored dots represent whether or not that gene contains orthologs within outgroup genomes, type II Accumulibacter genomes other than the IIC-UW6 genome, type I Accumulibacter genomes, or only clade IA genomes.

We finally characterized the expression dynamics of flexible gene content in strain IA-UW4 (Fig. 6). Since a majority of the differentially expressed flexible genes in strain IIC-UW6 were annotated as hypothetical proteins, we focused on putative differentiating features of clade IA-UW4. Flexible gene content for IA-UW4 can be defined as any ortholog that is not present in the IIC-UW6 genome, whether or not that ortholog is contained only within clade IA genomes, in type I as a whole, within other type II or clade IIC genomes, or in the *Dechloromonas* or *Rhodocyclus* outgroups. Thus, the absence of a particular ortholog in the strain IIC-UW6 genome but its presence in the IA-UW4 genome or any other genomes was inferred as a lack of functional capability of IIC-UW6 in this particular ecosystem.

Particularly striking are the differential expression profiles of nitrogen-cycling genes that do not contain orthologs in the IIC-UW6 genome. As stated above, clade IA-UW4 contains the *nap* system, whereas strain IIC-UW6 contains the *nar* system for reducing nitrate to nitrite (Fig. 4C). In strain IA-UW4, the *napDGH* subunits are differentially expressed between the anaerobic and aerobic phases, and these genes are present in the outgroups as well as both Accumulibacter types except the IIC-UW6 genome (Fig. 6). Both genomes contain a *nirS* nitrite reductase, but the respective homologs are not within the same COG, and thus, strain IA-UW4 contains a different *nirS* that is upregulated in the anaerobic phase. Strain IIC-UW6 does not contain both subunits of the nitric oxide reductase *norQD*, whereas strain IA-UW4 does. These subunits are not differentially expressed above the set threshold but exhibit the highest expression levels upon acetate contact in the anaerobic phase (Fig. 4B). Additionally, strain IIC-UW6 does not contain the nitrous oxide reductase gene *nosZ* (Fig. 4B), whereas strain IA-UW4 does, and this is differentially expressed in the anaerobic phase (Fig. 6). These results suggest that strain IA-UW4 may have the capability for full denitrification from nitrate to nitrogen gas or at least use the reduction of nitrate/nitrite as a source of energy in the anaerobic phase in a denitrifying bioreactor. Although strain IIC-UW6 contains the *nirBD* nitrite reductase complex for reducing nitrite to ammonium, these genes are uniformly expressed throughout the cycle (Fig. 4B).

## DISCUSSION

In this work, we performed time series transcriptomics to explore the gene expression profiles of Accumulibacter strains belonging to clades IA and IIC, which coexisted within the same bioreactor ecosystem. By applying genome-resolved metagenomic and metatranscriptomic techniques, we were able to postulate niche-differentiating features of distinct Accumulibacter clades based on core and accessory gene contents and differential expression profiles. We assembled high-quality genomes from four Accumulibacter clades and used long reads to generate a single contiguous scaffold of the IIC-UW6 genome. By performing an updated clustering of orthologous groups of genes analysis, we were able to better demonstrate the similarity and uniqueness among Accumulibacter strains from this system. Unexpectedly, although clade IA dominated in abundance by *ppk1* locus quantification, clade IIC was more transcriptionally active. By comparing the expression profiles of COGs shared between strains IA-UW4 and IIC-UW6, we found that genes involved in EBPR-related pathways may differ between the strains with respect to transcription-level regulation. Notably, strain IIC-UW6 showed a strong upregulation of the high-affinity Pst phosphorus transport system in the aerobic phase. The high transcription levels of the Pst system in IIC-UW6 toward the end of the aerobic phase when phosphorus concentrations were lower suggest that the IIC-UW6 strain may be more sensitive to bulk phosphate concentrations. This would be congruent with specialization to relatively low-phosphorus conditions, whereas the lack of differential expression of the Pst system in IA-UW4 suggests that this strain is more specialized under high-phosphorus conditions. Strain IA-UW4 contains most of the subunits for the individual key steps for denitrification and exhibits strong anaerobic upregulation of these genes, suggesting a differentiating role for strain IA-UW4 in nitrogen cycling under denitrifying conditions. Additionally, the IA-UW4 strain contains all subunits for encoding the Hox anaerobic hydrogenase system, which exhibits upregulated gene expression in the anaerobic phase. However, the IIC-UW6 strain is missing one of the subunits, even though the other subunits were expressed (but not differentially). The production of hydrogen gas could be a differentiating role for the IA-UW4 strain, as this would impact potential interactions with diverse flanking community members in addition to replenishing NAD^+^ (13, 24, 39).

The Accumulibacter lineage harbors extensive diversity between and among individual clades, which is exhibited not only by *ppk1-*based phylogenies but also by pairwise genome-wide ANI and COG comparisons. For example, strain IIA-UW1 and strain IA-UW3 were present in the same bioreactor ecosystem and share only 85% ANI (22). In the bioreactor enrichment from this study, the dominant strains IA-UW4 and IIC-UW6 share less than 80% ANI (Fig. 2B). Recently, a cutoff of >95% ANI has been suggested to delineate distinct bacterial species or cohesive genetic units (40), which is based on observations of coverage gaps at 90 to 95% sequence identity when metagenomic reads were mapped back to assembled MAGs (41–44). These sequence cutoffs have also been benchmarked against homologous recombination rates and ratios of nonsynonymous to synonymous (*dN*/*dS*) nucleotide differences (45). Among Accumulibacter genomes adhering to the established *ppk1*-based phylogeny, genomes within individual clades fall within the >95% ANI threshold, whereas ANI comparisons within and between types I and II are more variable (Fig. 2B). Genomes within type I are similar by approximately 90% ANI, whereas genomes within type II are not as coherent.

The apparent coexistence of diverse Accumulibacter clades within an ecosystem then raises questions of ecological roles, putative interactions, and evolutionary dynamics of this lineage over time and space. The ecotype model of speciation suggests that cohesive, ecologically distinct ecotypes can coexist because they occupy separate niche spaces (3, 46). Because Accumulibacter has not been isolated in pure culture to date, most ecological and evolutionary inferences have been made from genome-based comparisons (26, 30) or by mapping metagenomic reads back to assembled genomes with minimum percent identity cutoffs that may not sufficiently distinguish among subclades (47, 48). However, the apparent diversity within the Accumulibacter lineage may require reevaluation of the phylogeny and taxonomy overall. Although the *ppk1*-based clade designations coincide with genome-wide ANI-based cutoffs remarkably well (see Fig. S2B in the supplemental material), there are some discrepancies. A few Accumulibacter genomes have been assembled that do not adhere to the *ppk1*-based population structure but fall within the Accumulibacter lineage based on a species tree of single-copy core genes (Fig. S2A), such as the proposed novel species “*Candidatus* Accumulibacter aalborgensis” (49). Additionally, a few genomes preliminarily classified by the Genome Taxonomy Database (GTDB) as Accumulibacter branch outside the established nomenclature and are quite divergent based on the species and *ppk1* phylogenies as well as genome-wide and *ppk1* sequence similarities (Fig. 2B; Fig. S2). Overall, substantial work is needed to reevaluate the phylogenetic structure of the Accumulibacter lineage as a whole and connect it with signatures of homologous recombination and evolutionary trajectories within coexisting clades to understand the genetic boundaries and, thus, the ecological interdependencies of individual clades or strains.

10.1128/mSystems.00474-21.2 FIG S2 (A) Species tree of all Accumulibacter reference genomes, UW-assembled genomes, and outgroup genomes using single-copy markers from GTDB-tk (73). A phylogenetic tree was constructed using RAxML with 100 rapid bootstraps and visualized in iTOL. (B) Pairwise BLAST percent identity scores for *ppk1* coding regions for each reference genome in Fig. 2B, in the order of the *ppk1* phylogeny in Fig. 2A. Raw pairwise percent identity scores are available at https://figshare.com/articles/dataset/Pairwise_Accumulibacter_ppk1_BLAST_Percent_D_Scores/13237247. Download FIG S2, TIF file, 0.5 MB. Copyright © 2021 McDaniel et al. 2021 McDaniel et al. https://creativecommons.org/licenses/by/4.0/ This content is distributed under the terms of the Creative Commons Attribution 4.0 International license.

Although the boundaries of individual Accumulibacter clades or strains have not been entirely resolved to infer separate ecotypes, denitrification capabilities of specific clades have been repeatedly suggested to be a niche-differentiating feature (14, 23, 25). Previously, two separate bioreactors fed with either acetate or propionate as the sole carbon source were enriched with different Accumulibacter morphotypes, which were also linked to different denitrification capabilities (28). A study with a bioreactor highly enriched in clade IC inferred the simultaneous use of oxygen, nitrite, and nitrate as electron acceptors under microaerobic conditions due to either metabolic flexibility or heterogeneity within clade IC members (23). The resident strain IC-UW-LDO also possessed the full suite of genes for denitrification and expressed them under microaerobic conditions (14). Coexisting Accumulibacter strains with differing denitrifying abilities have also been observed in a denitrifying EBPR bioreactor enriched in clade IA and clade IC (17, 27). The clade IA strain carried genes for complete denitrification, whereas the co-occurring clade IC strain and other flanking community members did not (27). Additionally, Gao et al. performed an ancestral genome reconstruction analysis similar to the one reported by Oyserman et al. (30) to specifically understand the flux of denitrification gene families (27). The *nir* gene cluster was identified as a core COG to all sampled Accumulibacter genomes, whereas the periplasmic nitrate reductase *napAGH* and nitrous oxide reductase *nosZDFL* clusters were core to only type I Accumulibacter genomes and inferred to have been lost for some type II genomes (27). Additionally, Wang et al. subsequently observed that an internal stop codon within the open reading frame of the *nosZ* gene in a clade IC strain coincided with decreased transcripts mapping back (17, 27). In our study, we observed differential expression of denitrification genes in strain IA-UW4 but not strain IIC-UW6. These expression differences could explain why the two strains can coexist and contribute to overall ecosystem functioning (e.g., phosphorus removal) by using different electron acceptors under anaerobic/aerobic conditions. Although the synthetic feed for the bioreactors in this study inhibits ammonia oxidation through allylthiourea addition (and, thus, inhibits nitrite/nitrate production to fuel respiratory denitrification), the expression profiles of denitrification genes in strain IA-UW4 suggest that this strain is equipped to have a differentiating role in a denitrifying bioreactor. We specifically observed differential expression of nitrate reductase and nitrous oxide reductase genes in strain IA-UW4, where homologs of these genes were not present in the strain IIC-UW6 genome and other type II genomes. From our work and previous studies, the lack of the complete genetic repertoire for denitrification among and within individual clades in addition to transcriptional-based evidence suggest that denitrification capabilities could be a niche-differentiating role for specific Accumulibacter populations.

Accumulibacter’s anaerobic metabolism has also been shown to differ under various substrate or stoichiometric conditions and possibly between the two Accumulibacter types. Recent simulations of ‘omics data sets paired with enzymatic assays hint that the routes of anaerobic metabolism for Accumulibacter may differ based on environmental conditions (36). Under high-acetate conditions, polyphosphate accumulation arises through the glyoxylate shunt, whereas under increased glycogen concentrations, glycolysis is used in conjunction with the reductive branch of the tricarboxylic acid (TCA) cycle (36). Furthermore, Welles et al. found that type II Accumulibacter can switch to partial glycogen degradation more quickly than type I, enabling type II to fuel VFA uptake under polyphosphate-limited conditions (50, 51). Conversely, when concentrations of polyphosphate are high, VFA uptake rates are higher in type I. However, these studies identified Accumulibacter lineages only based on *ppk1* types and therefore extrapolated metabolic assumptions from presumably more resolved strains to the entire type. These broad interpretations may obscure apparent functional differences between the two types and individual clades. Future experiments could integrate multi-‘omics approaches to investigate not only metabolic flexibility under different substrate conditions and perturbations but also how metabolisms are distributed among Accumulibacter clades in bioreactor ecosystems that are not completely enriched in a single clade or strain and elucidate key functional differences.

### Conclusions.

In this study, we integrated genome-resolved metagenomics and time series metatranscriptomics to understand gene expression patterns of coexisting Accumulibacter strains within a bioreactor ecosystem. We found evidence for denitrification gene expression in the dominating IA-UW4 strain but higher overall transcriptional activity for the IIC-UW6 strain along with differential expression of a high-affinity phosphorus transporter. The coexistence of Accumulibacter populations exhibiting different gene expression patterns for phosphorus and nitrogen cycling and hydrogen productions suggests niche-differentiating features and contributions to different metabolic programs. Detailed experiments incorporating microautoradiography combined with fluorescence *in situ* hybridization (MAR-FISH) could be applied to confirm the metabolic activities of coexisting Accumulibacter populations and how they contribute to important EBPR-related functions (52). These results also highlight the need to reevaluate the phylogenetic structure of the Accumulibacter lineage and define how potential genetic boundaries may translate to individual ecological niches that allow for the coexistence of multiple clades, species-like groups, or strains. Additionally, we highlight an approach for exploring functions of coexisting species-like uncultivated microbial lineages by exploring gene expression patterns of shared and accessory genes. Overall, this work emphasizes the need to understand the basic ecology and evolution of microorganisms underpinning biotechnological processes, which can lead to better treatment process control and resource recovery outcomes in the future (53).

## MATERIALS AND METHODS

### Bioreactor operation and sample collection.

A laboratory-scale sequence batch reactor (SBR) was seeded with activated sludge from the Nine Springs Wastewater Treatment Plant (Madison, WI, USA) during August 2015. The bioreactor was operated to simulate EBPR as described previously (24). Briefly, an SBR with a 2-liter working volume was operated under established biphasic feast-famine conditions: a 6-h cycle consisting of the anaerobic phase (sparging with N_2_ gas) for 110 min, the aerobic phase (sparging with oxygen) for 180 min, a settling phase for 30 min, and drawing and feeding with an acetate-containing synthetic wastewater feed solution for 40 min. A reactor hydraulic residence time of 12 h was maintained by withdrawing 50% of the reactor contents after the settling phase and then filling the reactor with fresh nutrient feed; a mean solids retention time of 4 days was maintained by withdrawing 25% of the mixed reactor contents each day immediately prior to a settling phase. Allylthiourea was added to the synthetic feed solution to inhibit ammonia oxidation and indirectly inhibit nitrification/denitrification.

Three biomass samples for metagenomic sequencing were collected in September 2015 by centrifuging 2 ml of mixed liquor at 8,000 × *g* for 2 min, and DNA was extracted using a phenol-chloroform extraction protocol. Seven samples for RNA sequencing were collected throughout a single EBPR cycle (3 in the anaerobic phase and 4 in the aerobic phase) in October 2016 according to the sampling strategy reported previously by Oyserman et al. (13). Samples were flash-frozen in liquid nitrogen immediately after centrifuging and discarding the supernatant. RNA was extracted using a TRIzol-based extraction method (Thermo Fisher Scientific, Waltham, MA) followed by phenol-chloroform separation and RNA precipitation. RNA was purified following on-column DNase digestion using the RNase-free DNase set (Qiagen, Venlo, The Netherlands) and cleaned up with the RNeasy minikit (Qiagen, Venlo, The Netherlands). Accumulibacter clade quantification was performed on biomass samples collected on the same day of the metatranscriptomics experiment using clade-specific *ppk1* qPCR primers as described previously by Camejo et al. (23).

### Metagenomic and metatranscriptomic library construction and sequencing.

Metagenomic libraries were prepared by shearing 100 ng of DNA to 550-bp-long products using the Covaris LE220 system and size selected with Spri beads (Beckman Coulter). The fragments were ligated with end repair, A-tailing, Illumina-compatible adapters (IDT Inc.) using the Kapa-Illumina library preparation kit (Kapa Biosystems). Libraries were quantified using the Kapa Biosystems next-generation sequencing library qPCR kit and run on a Roche LightCycler 480 real-time PCR instrument. The quantified libraries were prepared for sequencing on the Illumina HiSeq platform using the v4 TruSeq paired-end cluster kit and the Illumina cBot instrument to create a clustered flow cell for sequencing. Shotgun sequencing was performed at the University of Wisconsin—Madison Biotechnology Center DNA Sequencing Facility on the Illumina HiSeq 2500 platform with the TruSeq SBS sequencing kits, followed by 2-by-150 indexing. Raw metagenomic data consisted of 219.4 million 300-bp Illumina HiSeq reads with approximately 3.9 Gbp per sample. Nanopore sequencing was performed according to the user’s manual, version SQK-LSK108.

Total RNA submitted to the University of Wisconsin—Madison Biotechnology Center was verified for purity and integrity using a NanoDrop 2000 spectrophotometer and an Agilent 2100 bioanalyzer, respectively. RNA-seq paired-end libraries were prepared using the TruSeq RNA library prep kit v2 (Illumina, San Diego, CA). Each sample was processed for ribosomal depletion using the Ribo-Zero rRNA removal kit (bacteria). mRNA was purified from total RNA using paramagnetic beads (Agencourt RNAClean XP beads; Beckman Coulter Inc., Brea, CA) and fragmented by heating in the presence of a divalent cation. The fragmented RNA was then converted to cDNA with reverse transcriptase using SuperScript II reverse transcriptase (Invitrogen, Carlsbad, CA, USA) with random hexamer priming, and the resultant double-stranded cDNA was purified. cDNA ends were repaired, adenylated at the 3′ ends, and then ligated to Illumina adapter sequences. The quality and quantity of the DNA were assessed using an Agilent DNA 1000 series chip assay and a Thermo Fisher Qubit dsDNA HS assay kit. Libraries were diluted to 2 nM, pooled in an equimolar ratio, and sequenced on the Illumina HiSeq 2500 platform using a single lane of paired-end, 100-bp sequencing.

### Metagenomic assembly and annotation.

We applied two different assembly and binning approaches to obtain high-quality genomes of the dominant Accumulibacter strains present in the reactors. For the first approach, Illumina unmerged reads were quality filtered and trimmed using Sickle software v1.33 (54). Reads were merged with FLASH v1.0.3 22 (55), with a mismatch value of ≤0.25 and a minimum of 10 overlapping bases from paired sequences, resulting in merged read lengths of 150 to 290 bp. FASTQ files were then converted to FASTA format using Seqtk software v1.0 (56). Metagenomic reads from all three samples were then coassembled using the Velvet assembler with a k-mer size of 65 bp, a minimum contig length of 200 bp, and a paired-end insert size of 300 bp (57). Metavelvet was used to improve the assembly generated by Velvet (58). Metagenomic contigs were then binned using Maxbin (59). This approach allowed us to assemble high-quality genomes of clades IIA and IIC, named UW5 and UW6, respectively. Long Nanopore reads were generated according to standard protocols, used to scaffold assembled contigs with MeDuSa (60), manually inspected to remove short contigs, and decontaminated using ProDeGe (61) and Anvi’o (62). Further scaffolding was performed on both of these genomes using Nanopore long reads using LINKS (63) and GapCloser (64).

Because this pipeline did not produce high-quality genomes of clades IA and IIF, we applied a different approach to assemble these genomes. All reads from each metagenomic sample were individually assembled and coassembled using metaSPAdes (65). Reads from each metagenome were mapped against each of the assemblies using bbmap with a 95% sequence identity cutoff to obtain differential coverage (66), and contigs were binned using MetaBat (67). Identical clusters of bins were dereplicated across assemblies using dRep (68) to obtain the best-quality genomes of both clades IA and IIF, named UW4 and UW7, respectively. All genomes were quality checked using CheckM (29), and functional annotations were assigned with Prokka and KofamKOALA (69, 70). We were unable to scaffold the genomes of UW4-IA and UW7-IIF with long Nanopore reads because of their low abundance at the time of metagenomic sequencing.

Each genome was assigned to a particular clade by comparing both the *ppk1* sequence identity and ANI to those of previously published Accumulibacter genomes. A *ppk1* database was created using sequences from previously published Accumulibacter references and clone sequences. We searched for the *ppk1* gene in our draft genomes using this database and aligned the corresponding hits with MAFFT (71). A phylogenetic tree of aligned *ppk1* gene sequences from Accumulibacter references, select clone sequences, and the outgroups Dechloromonas aromatica RCB and Rhodocyclus tenuis DSM 110 was constructed with RAxML v8.1 with 100 rapid bootstraps (72). To use any given Accumulibacter reference genome in downstream comparisons, a confident *ppk1* hit had to be identified using the above-described methods. As a technical note, the IA-UW2 strain assembled previously by Flowers et al. (22) was renamed UW3, as the original assembly contained an additional contig that was likely from a prophage. The prophage contig was removed from the IA-UW2 strain and renamed UW3, and therefore, the UW numerical nomenclature follows in sequential order after UW3. A phylogenetic tree of *ppk1* nucleotide sequences was constructed and compared to select clone sequences. Pairwise genome-wide ANI was calculated among the four Accumulibacter draft genomes and all available Accumulibacter genomes in GenBank with FastANI (40). A species tree of all Accumulibacter reference and outgroup genomes was constructed using single-copy markers from GTDBK-tk (73), aligned with MAFFT (71), built with RAxML with 100 rapid bootstraps (72), and visualized in iTOL (74). To compare genome-wide ANI scores of Accumulibacter references with the pairwise nucleotide identities of the *ppk1* locus of all references, we performed pairwise BLAST analysis for all *ppk1* coding regions and reported the percent identity (75).

### Identification of core and accessory gene contents.

We identified core and accessory gene contents of Accumulibacter clades through clustering of orthologous groups of genes (COGs) using PyParanoid (76). We used the four Accumulibacter MAGs generated in this study (Table 1) and high-quality Accumulibacter reference genomes (>90% completeness and <5% redundancy) for this analysis, with Dechloromonas aromatica RCB and Rhodocyclus tenuis DSM 110 as outgroups (Accumulibacter references are listed in the table available at https://figshare.com/articles/dataset/Accumulibacter_References_Metadata/13237148). Briefly, an all-versus-all comparison of proteins for all genomes was performed using DIAMOND (77), pairwise homology scores were calculated with the InParanoid algorithm (78), gene families were constructed with MCL (79), and hidden Markov models (HMMs) were built for each gene family with HMMER (80). A phylogenetic tree was constructed for genomes used for the COG analysis by using the coding regions of the *ppk1* locus and overlaid with the presence/absence of core and flexible gene contents using the ete3 python package (81). A similarity score between UW Accumulibacter genomes was calculated as the pairwise Pearson correlation coefficient between gene families using the numpy python package (82). The presence and absence of different gene families were visualized in an upset plot between UW Accumulibacter genomes using the ComplexUpset package based on the UpSetR package in R (83). All data wrangling and plotting were performed using the tidyverse suite of packages in R (84).

### Metatranscriptomics mapping and processing.

Metatranscriptomics reads from each of the seven samples were quality filtered using fastp (85), and rRNA was removed with SortMeRNA (86). The coding regions of all four Accumulibacter genomes assembled in this study (Table 1) were predicted with Prodigal (87), annotated with both Prokka and KofamKOALA (69, 70), and concatenated together to create a mapping index. Metatranscriptomic reads were competitively pseudoaligned to the reference index, and counts were quantified with kallisto (88). Genes with a sum of more than 10,000 counts across all 7 samples were removed, as these are likely rRNAs that were not removed previously. Additionally, genes annotated as rRNAs by barrnap were manually removed (69).

To explore genes and metabolic functions that may exhibit hallmark anaerobic-aerobic feast-famine cycling patterns among the dominant IA-UW4 and IIC-UW6 strains, we identified sets of genes that were differentially expressed between anaerobic and aerobic conditions using DESeq2 (89). Differential gene expression calculations were performed for each strain separately to account for differences in the relative abundances of metatranscriptomic reads mapping back to each of the four MAGs. Low-count genes were removed individually for each genome by requiring that all genes had to have more than 10 counts mapped across 3 or more samples. For IA-UW4, 3,549 genes out of a total of 3,919 predicted genes remained after applying this filter, and for IIC-UW6, 4,297 genes out of a total of 4,877 predicted genes remained after applying this filter. We analyzed expression patterns of differentially expressed genes using a threshold cutoff of a ±1.5-log_2_ fold change (corresponding to an adjusted *P* value of 0.10) between anaerobic and aerobic conditions and then identified differentially expressed genes as core or accessory genes among clades IA (strain UW4) and IIC (strain UW6). For the core group analysis, the corresponding ortholog in each genome had to meet the minimum count threshold as described above to be included in the comparison, with genes in one or both of the strains being differentially expressed. For the accessory analysis for each strain, the gene had to be differentially expressed and not contained in the other genome but could be present in other strains or other genomes belonging to that clade.

### Data availability.

Raw sequencing files for the three metagenomes and seven metatranscriptomes and genome assemblies for the four Accumulibacter genomes are available in the NCBI database under BioProject accession number PRJNA668760. All genome assemblies used in this study in their current forms as well as transcriptomic count tables are available at https://figshare.com/projects/Metabolic_Plasticity_of_Accumulibacter_Clades/90614. All code is available at https://github.com/elizabethmcd/R3R4.
