# Supplementary figures and images for: Metabolic Differentiation of Co-occurring Accumulibacter Clades Revealed through Genome-Resolved Metatranscriptomics
Source: mSystems. 2021 Jul 6;6(4):e00474-21. doi: 10.1128/mSystems.00474-21 (PMC8407102; doi:10.1128/mSystems.00474-21)

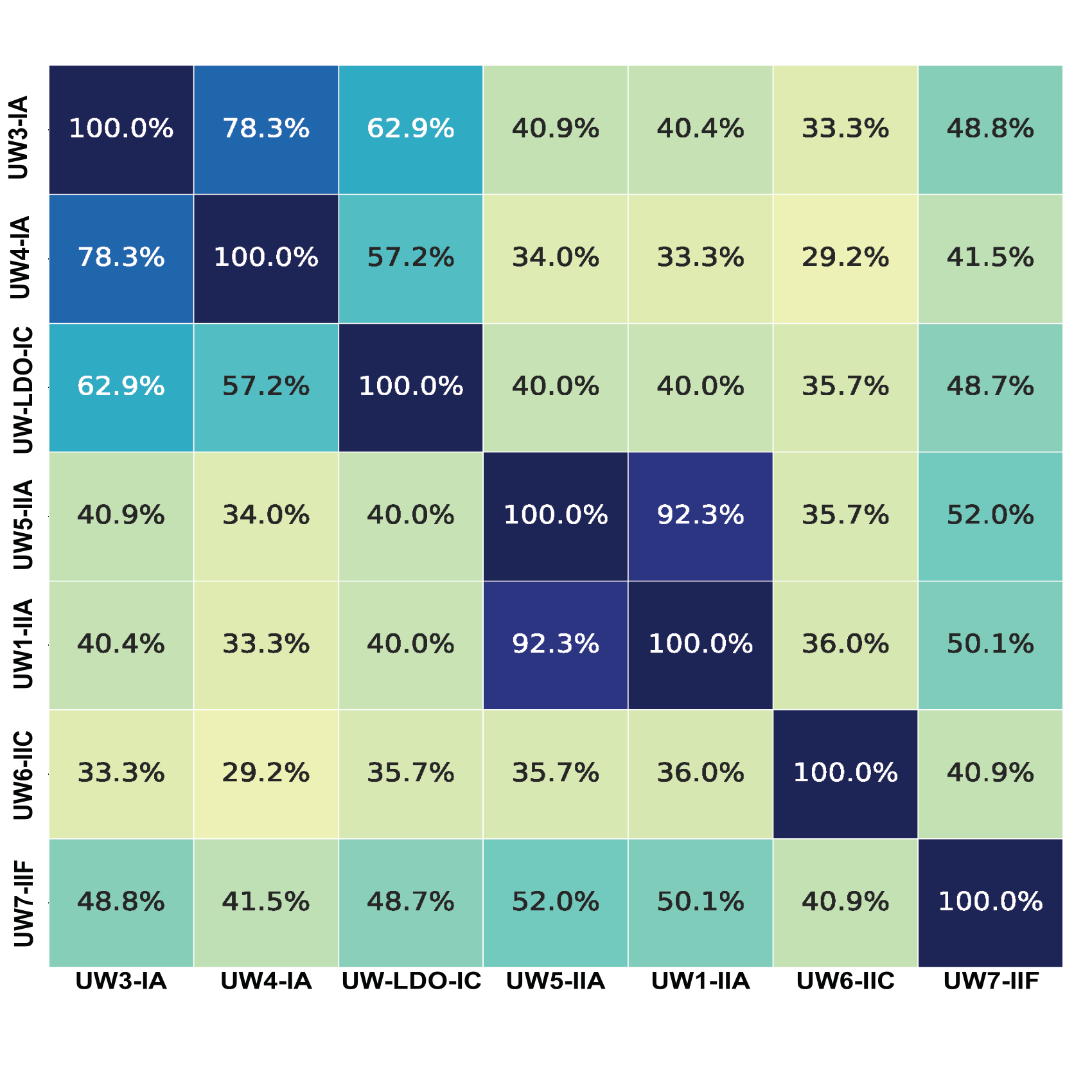

Supplement: FIG S1 [file msystems.00474-21-sf001.tif]

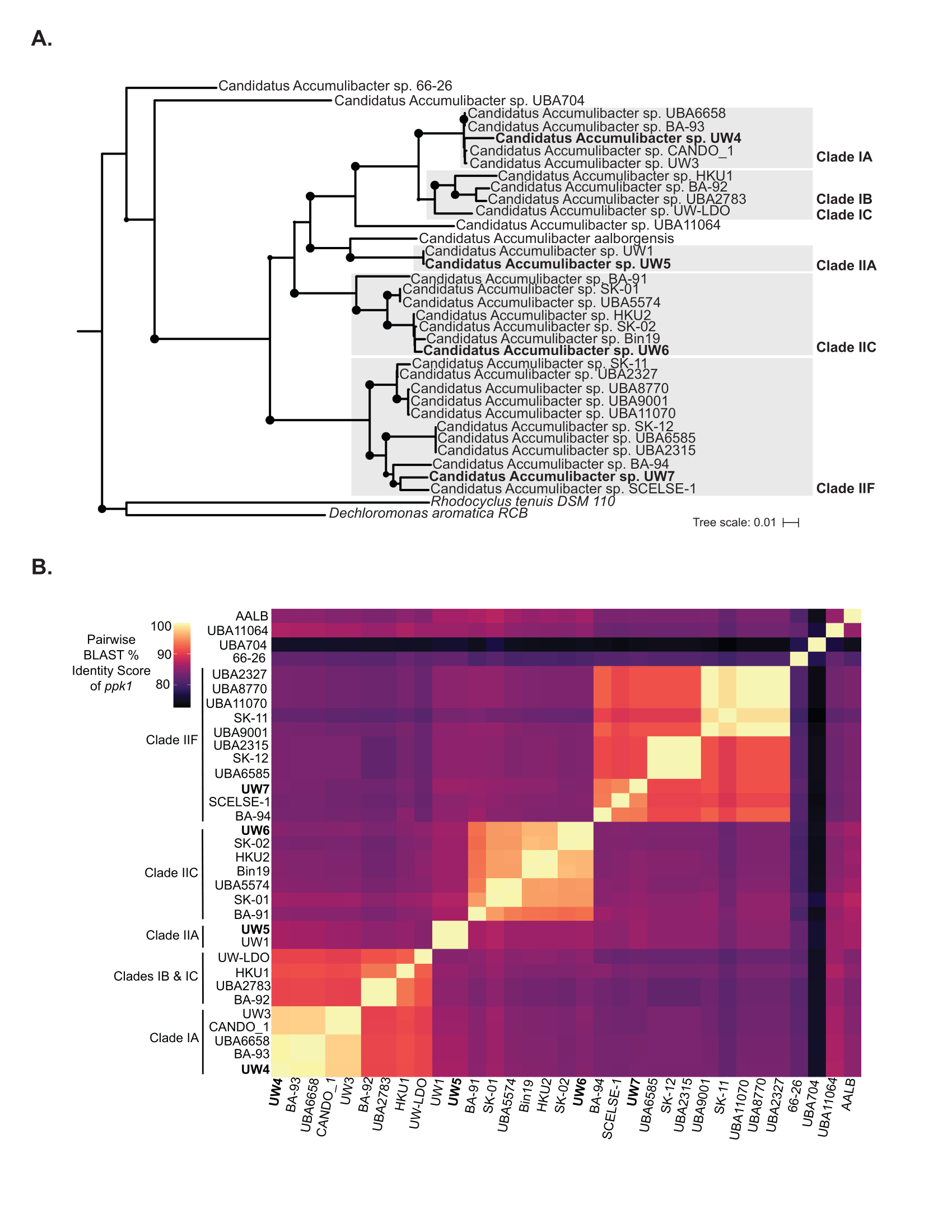

Supplement: FIG S2 [file msystems.00474-21-sf002.tif]
